# A novel approach to triple-negative breast cancer molecular classification reveals a luminal immune-positive subgroup with good prognoses

**DOI:** 10.1038/s41598-018-38364-y

**Published:** 2019-02-07

**Authors:** Guillermo Prado-Vázquez, Angelo Gámez-Pozo, Lucía Trilla-Fuertes, Jorge M. Arevalillo, Andrea Zapater-Moros, María Ferrer-Gómez, Mariana Díaz-Almirón, Rocío López-Vacas, Hilario Navarro, Paloma Maín, Jaime Feliú, Pilar Zamora, Enrique Espinosa, Juan Ángel Fresno Vara

**Affiliations:** 1 Molecular Oncology & Pathology Lab, INGEMM, La Paz University Hospital Health Research Institute-IdiPAZ, Madrid, Spain; 2 R&D department, Biomedica Molecular Medicine SL, Madrid, Spain; 3 Biostatistics Unit, La Paz University Hospital Health Research Institute-IdiPAZ, Madrid, Spain; 4 Department of Statistics, Operational Research and Numerical Analysis, National University of Distance Education (UNED), Madrid, Spain; 5 Department of Statistics and Operations Research, Faculty of Mathematics, Complutense University of Madrid, Madrid, Spain; 6 Medical Oncology Service, La Paz University Hospital Health Research Institute-IdiPAZ, Madrid, Spain; 7 Biomedical Research Networking Center on Oncology-CIBERONC, ISCIII, Madrid, Spain

## Abstract

Triple-negative breast cancer is a heterogeneous disease characterized by a lack of hormonal receptors and HER2 overexpression. It is the only breast cancer subgroup that does not benefit from targeted therapies, and its prognosis is poor. Several studies have developed specific molecular classifications for triple-negative breast cancer. However, these molecular subtypes have had little impact in the clinical setting. Gene expression data and clinical information from 494 triple-negative breast tumors were obtained from public databases. First, a probabilistic graphical model approach to associate gene expression profiles was performed. Then, sparse k-means was used to establish a new molecular classification. Results were then verified in a second database including 153 triple-negative breast tumors treated with neoadjuvant chemotherapy. Clinical and gene expression data from 494 triple-negative breast tumors were analyzed. Tumors in the dataset were divided into four subgroups (luminal-androgen receptor expressing, basal, claudin-low and claudin-high), using the cancer stem cell hypothesis as reference. These four subgroups were defined and characterized through hierarchical clustering and probabilistic graphical models and compared with previously defined classifications. In addition, two subgroups related to immune activity were defined. This immune activity showed prognostic value in the whole cohort and in the luminal subgroup. The claudin-high subgroup showed poor response to neoadjuvant chemotherapy. Through a novel analytical approach we proved that there are at least two independent sources of biological information: cellular and immune. Thus, we developed two different and overlapping triple-negative breast cancer classifications and showed that the luminal immune-positive subgroup had better prognoses than the luminal immune-negative. Finally, this work paves the way for using the defined classifications as predictive features in the neoadjuvant scenario.

## Introduction

Breast cancer (BC) causes 450,000 deaths every year worldwide^1^. BC is clinically and genetically heterogeneous^2^, and this heterogeneity has led to subdivisions in an attempt to treat patients more efficiently. The classical categorization considers the expression of hormonal receptors (estrogen receptors [ERs], and progesterone receptors [PRs]) and human epidermal growth factor receptor 2 (HER2) expression, because this determines the possibility of treatment with hormones and anti-HER2 therapies, respectively.

Triple-negative breast cancer (TNBC) is defined by a lack of ER and PR expression and a lack of HER2 overexpression. TNBC comprises a heterogeneous group of tumors. In 2000, Perou *et al*. proposed a classification of BC based on gene expression patterns. Most triple-negative tumors are included in the so-called basal-like molecular subgroup^3^, although both categories have up to 30% discordance^4^.

Several studies have developed specific molecular classifications for TNBC. For example, Rody *et al*. defined metagenes that distinguished molecular subsets within the group^5^. Lehmann *et al*. identified seven molecular subgroups: unstable; basal-like 1; basal-like 2; immunomodulatory; mesenchymal (MES)-like; mesenchymal stem-like (MSL); and luminal androgen receptor (LAR)^6^. The Immunomodulatory and MSL subtypes have recently been refined^7^. Burstein *et al*. applied non-negative matrix factorization and defined four subgroups: basal-like immune active; basal-like immune suppressed; mesenchymal; and luminal AR^8^. Other classifications have also been proposed by Sabatier^9^, Prat^10^, Jézéquel^11^, and Milioli^12^. Despite these extensive studies, the designation of TNBC molecular subtypes has had little impact in the clinical setting.

The so-called cancer stem cell hypothesis could provide a different way to categorize BC. It theorizes that cancer derives from a stem cell compartment that undergoes an abnormal and poorly regulated process of organogenesis analogous to many aspects of normal stem cells^13–15^. Depending on the activation point of these cancer stem cells, tumors will have varying characteristics. Poorly differentiated breast tumors would arise from the most primitive stem cells^14^. This hypothesis contextualizes BC molecular groups^1^ in a development framework. Moreover, molecular characterization of the claudin (CLDN)-low subtype reveals that these tumors are significantly enriched in epithelial-mesenchymal transition and stem cell-like features, while showing a low expression of luminal and proliferation-associated genes^16^.

In the present study, we applied probabilistic graphical models to a previously published TNBC cohort^5^. This technique allows exploring the molecular information from a functional perspective. Our aim was to tackle the molecular analysis of TNBC from a broad perspective, such as the cancer stem cell hypothesis, to provide a classification with clearer clinical implications.

## Methods

### TNBC gene expression and clinical data

Gene expression data from TNBC tumors and available clinical follow-up information were obtained from GSE31519. Gene expression values were magnitude normalized, and then log_2_ was calculated. The *Limma* R package^17^ was applied to avoid the batch effect. Finally, the complete dataset was mean centered. The probe with the highest variance of each gene within all patients was selected. The results obtained with the first database were then applied to a second database of patients treated with neoadjuvant chemotherapy, GSE25066. GSE25066 data was magnitude normalized and log_2_ was calculated just as with GSE31519.

### Probabilistic graphical model analysis

A probabilistic graphical model compatible with a high-dimensionality approach to associate gene expression profiles, including the most variable 2000 genes, was performed as previously described^18^. Briefly, the resulting network, in which each node represents an individual gene, was split into several branches to identify functional structures within the network. Then, we used gene ontology analyses to investigate which function or functions were overrepresented in each branch, using the functional annotation chart tool provided by DAVID 6.8 beta^19^. We used “homo sapiens” as a background list and selected only GOTERM-DIRECT gene ontology categories and Biocarta and KEGG pathways. Functional nodes were composed of nodes presenting a gene ontology enriched category. To measure the functional activity of each functional node, the mean expression of all the genes included in one branch related to a concrete function was calculated. Differences in functional node activity were assessed by class comparison analyses. Finally, metanodes were defined as groups of related functional nodes using nonsupervised hierarchical clustering analyses.

### Sparse k-means classification

Sparse k-means was used to establish the optimal number of tumor groups. This method uses the genes included in each node and metanode, as previously described^20^. Briefly, classification consistency was tested using random forest. An analysis using the consensus clustering algorithm^21^ as applied to the data containing the variables that were selected by the sparse K-means method^22^ has provided an optimum classification into two subtypes in previous studies^20^. In order to transfer the newly defined classification from the main dataset to other datasets, we constructed centroids for each defined subgroup, using genes included in various metanodes.

### Assignation to groups defined by other molecular classifications

Tumors in the main dataset were assigned to a single group according to previously defined molecular classifications: PAM50 + CLDN low was assigned using the single sample predictor^10^. Burstein’s four subtypes were assigned using an 80-gene signature^8^. The TNBC4 type was performed in two steps: first, Lehmann’s seven subtypes were assigned using centroids constructed from 77 tumors included in the dataset that was previously assigned, and then Immunomodulatory and MSL groups were redefined as previously described^7^.

### Statistical analyses and software suites

Survival curves were estimated using Kaplan–Meier analyses and compared with the log-rank test, using relapse free survival (RFS) as the end point. RFS was defined as the time between the day of surgery and the date of distant relapse or last date of follow-up. Correlations were assessed using Pearson’s r and linear regression. Differences in functional node activity between groups were assessed by the Kruskal–Wallis test, and multiple comparisons were assessed using the Dunn’s multiple comparisons test. Box-and-whisker plots are Tukey boxplots. All p-values were two-sided, and P < 0.05 was considered statistically significant. Expression data and network analyses were performed in MeV and Cytoscape software suites^23^. The SPSS v16 software package, GraphPad Prism 5.0 and R v2.15.2 (with the Design software package 0.2.3) were used for all the statistical analyses.

## Results

### Gene expression and clinical data

Gene expression data and clinical information from 579 TNBC tumors were obtained from GSE31519. Some 85 samples were excluded because the patients had been treated with neoadjuvant chemotherapy or a different platform had been used. As a consequence, the data from 494 TNBC tumors from GSE31519 were used in subsequent analyses. Gene expression was normalized, the batch effect was corrected and the most variant probe was selected for each gene. The resulting dataset, including expression values from 13,146 genes will be referred to as the main dataset from now on.

Gene expression data from 508 breast cancer samples treated with neoadjuvant taxane-anthracycline chemotherapy were retrieved from GSE25066. A total 153 of these 508 samples were identified as TNBC.

### Clinical features

All available clinical features of the main dataset and the neoadjuvant dataset are presented in Table 1. The main dataset’s population of tumors tended to be large (>T1 in 56% of the population), poorly differentiated (G3 in 57% of the samples), with no node invasion (N0 in 51% of the samples) and most of the patients were not treated with adjuvant chemotherapy (52%). The neoadjuvant dataset’s population of tumors tended to be T2 (44%) and T3 (32%), poorly differentiated (G3 in 81% of the samples), and N1 (46%) with 32% of the patients achieving a complete pathological response after neoadjuvant treatment.

**Table 1:** Clinical features of the main and neoadjuvant datasets.

	Main Dataset	Neoadjuvant dataset	p-value
Number of patients	494	153	
**Tumor Size**			
T1	99 (20%)	9 (6%)	<0.0001
>T1	276 (56%)	144 (94%)	
NA	119 (24%)		
**Tumor Grade**			
G1&2	103 (21%)	16 (10%)	0.0001
G3	280 (57%)	124 (81%)	
NA	111 (22%)		
**Lymph node status**			
N0	251 (51%)	37 (24%)	<0.0001
N1	68 (14%)	116 (76%)	
NA	175 (35%)		
**Adjuvant Chemotherapy**			
No	257 (52%)		
Yes	71 (14%)		
NA	166 (34%)		
**Pathological Response**			
RD		95 (62%)	
pCR		53 (34%)	

Size data is divided into T1 (<2 cm) and >T1 (>2 cm) tumors; grade is classified as G1&2 (well or moderately differentiated tumors) or G3 (poorly differentiated tumors); lymph node status represents lymph node invasion (N0: no invasion; N1: invasion or metastasis); and the adjuvant chemotherapy column comprises patients who had been treated with adjuvant chemotherapy or not. The pathological response column stands for the response to neoadjuvant treatment (RD: residual disease; pCR: pathological complete response). The chi-squared test confirmed that both cohorts are different regarding clinical parameters and treatment.

### Molecular characterization of TNBC

**Figure 1:**
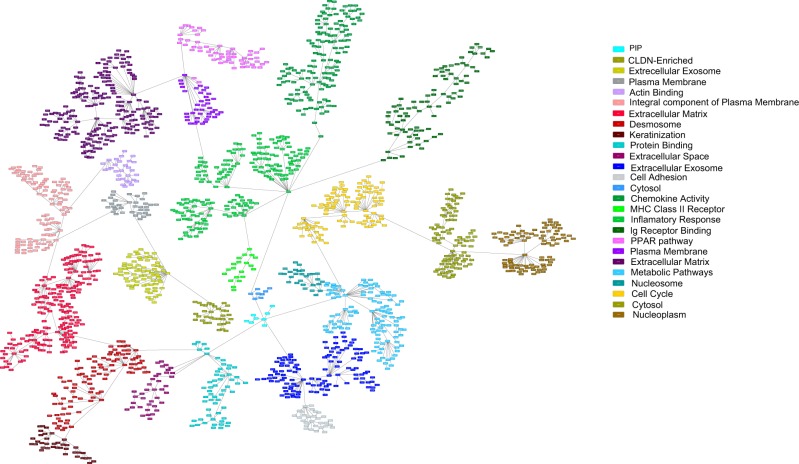
PGM resulting network; each functional node is encoded from 0 to 26. Each box (node) represents one gene, and lines (edges) connect genes with related expression. Functional nodes are represented by the same color, and metanodes are presented the same color palette, with basal nodes in red, luminal nodes in blue and immune nodes in green.

A gene expression-based network, including the 2000 most variant genes in the development dataset, was constructed using a probabilistic graphical model (PGM) (Fig. 1). The functional structure of the network was explored using gene ontology analyses, and 26 functional nodes were defined (Fig. 1 and Sup. File 1). Functional node activity was calculated and relationships between nodes were assessed using a hierarchical clustering (HCL) analysis (Sup. File 2). Functional node 1 is composed of 34 genes, including the CLDN3, CLDN4 and CLDN7 genes. On the other hand, functional nodes 15 (chemokine activity), 16 (major histocompatibility complex class II receptor activity), 17 (immune response) and 18 (antigen binding) were related to various aspects of the immune response and clustered together as an “immune metanode” in the HCL analysis (Sup. File 2). Additionally, functional node 19 contained genes related to the peroxisome proliferator-activated receptor (PPAR) signaling pathway, and functional node 24 contained genes involved in the G1/S transition of mitotic cell cycle (Sup. File 1).

**Figure 2:**
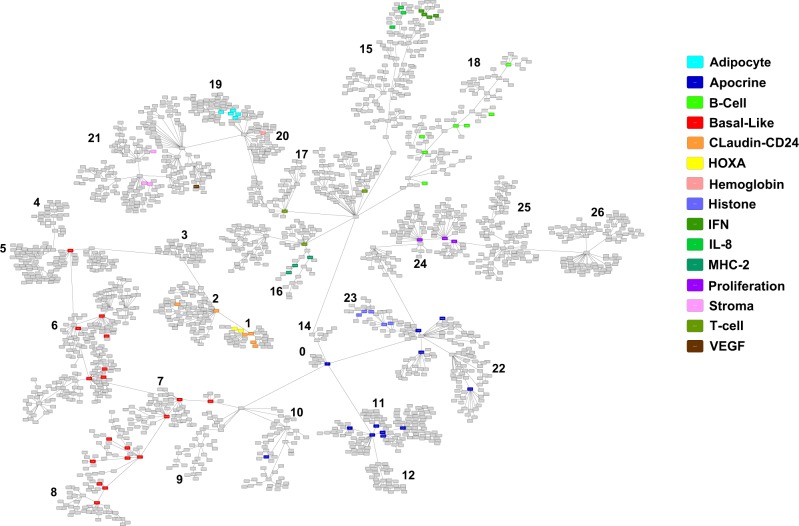
PGM represents the resulting network in which each functional node is encoded from 0 to 26, each box (node) represents one gene and lines (edges) connect genes with related expression. Genes from Rody’s metagenes are represented by different colors.

We then used the method described by Rody *et al*. to assess 15 metagenes (series of genes known to be related to one specific biological function or characteristic)^5^. Genes within a given metagene appeared close to each other in our network. Additionally, related metagenes, i.e., B-cell and IL-8 metagenes, also appeared close to each other (Fig. 2).

Functional nodes 5, 6, 7, 8 and 10 in our network had different gene ontologies related to an integral component of the plasma membrane, extracellular matrix, desmosomes, keratinization, and extracellular space, respectively. However, these five nodes appeared to correlate in the HCL analysis (Sup. File 2) and included genes from Rody’s basal-like metagene (Fig. 2). Thus, from now on, these five functional nodes were grouped as the basal metanode (Fig. 1). In the same way, functional nodes 0, 9, 11, 14, 22 and 23 were related to protein binding, extracellular exosomes, sequence-specific DNA binding, metabolic pathways and nucleosomes, respectively, again grouped together in the HCL analysis and including genes from Rody’s apocrine/luminal metagene, so they were defined as the luminal metanode (Fig. 1).

### Cellular classification

**Figure 3:**
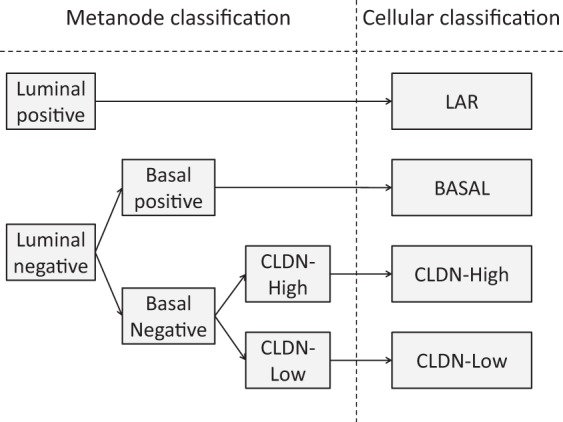
Workflow from the sparse k-means groups in each metanode to the final cellular classification.

The sparse k-means method was used to group samples into a limited number of clusters based on functional nodes and metanodes. Samples from the basal and luminal metanodes and the CLDN-enriched functional node were each divided into two groups. Mimicking the cancer stem cell hypothesis, we established the following workflow (Fig. 3): Samples with high luminal metanode activity were classified as the luminal androgen receptor group (LAR). Tumors showing low luminal metanode activity and high basal metanode activity from the basal subgroup were classified as basal. Finally, tumors with low activity in both the basal and luminal metanodes were screened for CLDN-enriched node expression. Samples showing low activity for the CLDN-enriched functional node were categorized as CLDN-low, whereas samples showing high activity for CLDN-enriched functional node were labeled as CLDN-high (Fig. 3).

From the 494 samples in the main dataset, the cellular classification defined 91 (18%) LAR, 53 (11%) CLDN-low, 310 (63%) basal and 40 (8%) CLDN-high samples. Only 7 (1.5%) samples showed high activity in both the luminal and basal metanodes (Table 2).

**Table 2:** Number of tumors classified in each metanode sparse k-means group and in the cellular classification.

Luminal	N	Basal	N	CLDN	Tumors	% of total	Cellular	N
—	403 (82%)	—	93 (23%)	High	40 (43%)	8%	CLDN-High	40 (8%)
				Low	53 (57%)	11%	CLDN-Low	53 (11%)
		+	310 (77%)	High	245 (79%)	50%	Basal	310 (63%)
				Low	65 (21%)	13%		
+	91 (18%)	—	84 (92%)	High	79 (94%)	16%	LAR	91 (18%)
				Low	5 (6%)	1%		
		+	7 (8%)	High	7 (100%)	1%		
				Low	0	0%		

Clinical characteristics from the various entities of cellular classification are shown in Table 3. Basal subtype tumors were mostly small-sized, poorly differentiated and without lymph node infiltration. The CLDN-high subtype tumors were large, had poor differentiation and no lymph node infiltration. The CLDN-low as well as the LAR tumors were large, more differentiated and showed more infiltration than the basal and CLDN-high tumors. Cellular classification does not show a significant relationship to RFS (Sup. File 3), nor did basal and luminal metanode activities show prognostic value. CLDN-high tumors showed a trend toward a poorer prognosis than CLDN-low, but again, the differences were not significant.

**Table 3:** Number of tumors with clinical characteristics.

Cellular Classification	Tumor size			Grade			Nodal		
	T1	>T1	p-value	G1 or G2	G3	p-value	N0	N1	p-value
Basal	76 (32%)	163 (68%)	0.169	45 (18%)	199 (82%)	0.015	168 (83%)	35 (17%)	0.262
CLDN-High	2 (7%)	27 (93%)	0.023	5 (14%)	31 (86%)	0.110	19 (83%)	4 (17%)	0.795
CLDN-Low	10 (24%)	32 (76%)	0.853	20 (49%)	21 (51%)	0.005	28 (72%)	11 (28%)	0.313
LAR	11 (17%)	54 (83%)	0.121	33 (53%)	29 (47%)	<0.001	36 (67%)	18 (33%)	0.056
Total	99 (26%)	276 (74%)	—	103 (27%)	280 (73%)	—	251 (79%)	68 (21%)	—

T1: tumor smaller than 2 cm; >T1: tumor larger than 2 cm; G3: grade 3; G1 or G2: grade 1 or grade 2; Nodal (N0): no node infiltration; N1: node infiltration. % is calculated using the total amount of a row for each clinical characteristic. Fisher exact test were performed between each group of the cellular classification and the total population (significant p-value = 0.05).

### Activity of functional nodes in cellular groups

The activity of the main functional nodes was assessed in each cellular group. CLDN-low tumors had lower activity than every other tumor subgroup in the functional nodes related to alpha-amylase activity and regulation of actin cytoskeleton, and higher activity than the other subgroups in the haptoglobin binding functional node. CLDN-high tumors had lower activity than basal tumors in the actin binding functional node, higher activity than tumors belonging to any other subgroup in chemokine activity functional node and lower activity than CLDN-low and LAR subtypes in the haptoglobin binding functional node. Basal tumors had higher activity than any other tumor in the functional nodes related to cell adhesion and regulation of the actin cytoskeleton. Finally, LAR tumors had lower activity in the nodes related to cell adhesion, G1/S transition of mitotic cell cycle and chemokine activity (Sup. File 4).

### Immune metanode activity: Immune characteristics

On the other hand, taking the immune metanode into account, tumors were split according to their immune (IM) activity. High/low immune activity was defined with the sparse K-means method using genes included in the IM metanode. Some 259 (52%) samples were included in the IM-positive (IM+) group and 235 (48%) were included in the IM-negative (IM−) group (Table 4).

**Table 4:** Immune characteristic interaction with cellular classification. According to the chi-squared test, IM characteristics and cellular classification are dependent.

IM negative			IM positive		
Cellular Classification	Tumors	%	Cellular Classification	Tumors	%
Basal	159	68%	Basal	151	58%
CLDN-Low	23	10%	CLDN-Low	30	12%
LAR	42	18%	LAR	49	19%
CLDN-High	11	5%	CLDN-High	29	11%

**Figure 4:**
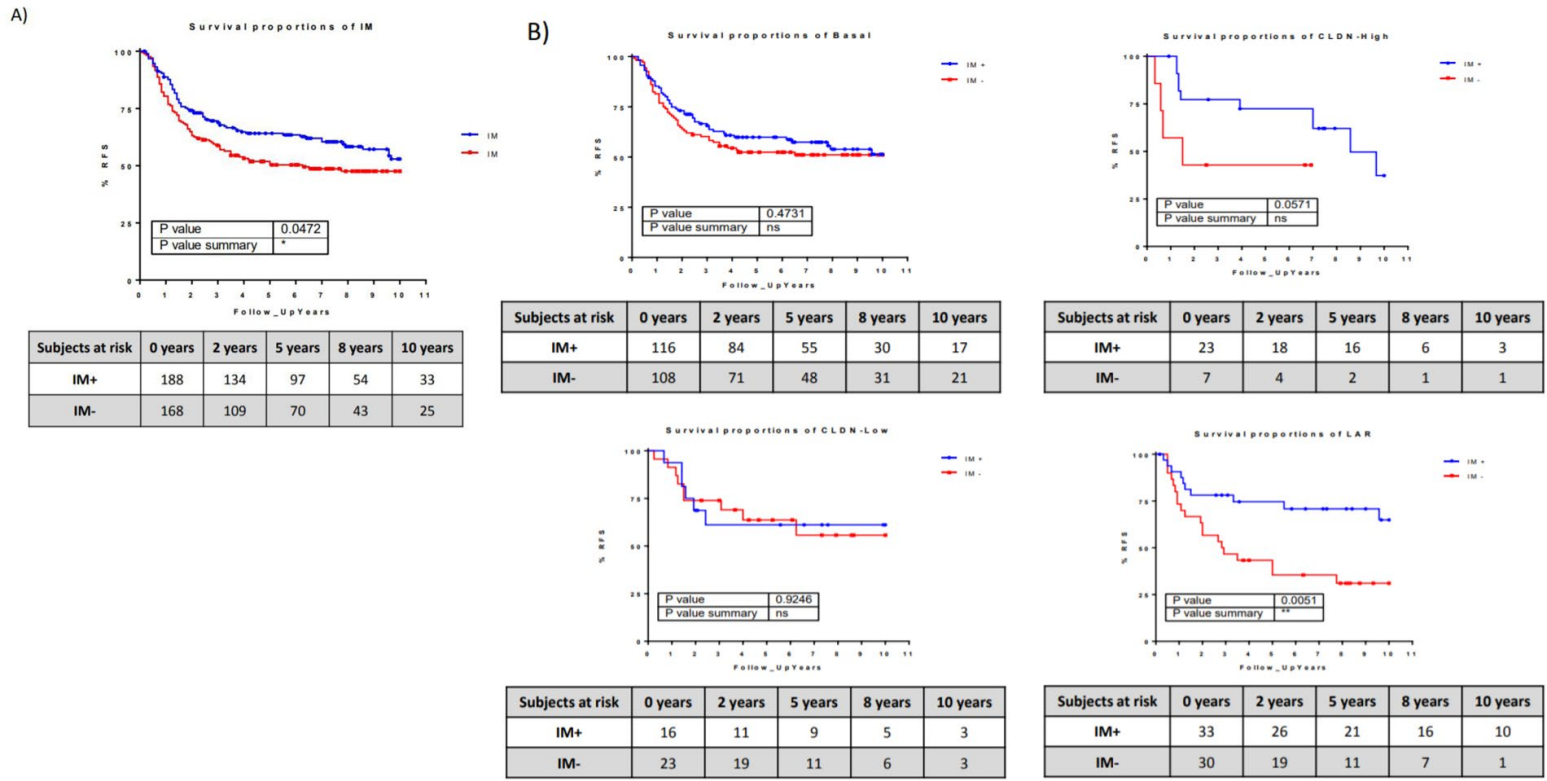
Kaplan-Meier survival curves represent the survival rate of immune-positive and immune-negative tumors in the whole cohort (**A**) and in the four cellular subgroups (**B**).

IM+ tumors had a better prognosis than IM- tumors (hazard ratio [HR], 0.7286; 95% confidence interval [CI] 0.5329–0.9961; P < 0.05) (Fig. 4A). In addition, the immune metanode activity had a prognostic impact on the groups defined by the cellular classification. Patients with IM+/LAR subtype tumors had a better prognosis than those with IM−/LAR tumors (HR, 0.3474; 95% CI 0.1657–0.7284; P < 0.05). Also, patients with IM+/CLDN-high tumors had a better prognosis than those with IM−/CLDN−, although these differences did not reach statistical significance (HR, 0.3556; 95% CI 0.04115–0.9828; P = 0.057). IM activity had no impact on the prognosis of the basal and CLDN-low subtypes (Fig. 4B).

### Comparison between Cellular classification and PAM50, TNBC4-type and Burstein’s classifications

**Figure 5:**
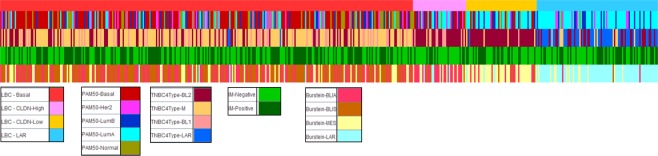
Various molecular classifications compared with the cellular classification. From top to bottom, cellular, PAM50 + CLDN-low, Lehmann 2016 TNBC4 type, immune and Burstein’s classifications are presented.

Cellular classification and previous classifications were compared (Fig. 5). The basal subtype is highly enriched in basal-like immune suppressed (BLIS) and basal-like immune associated (BLIA) (Burstein 2015), basal (PAM50 + CLDN-low) and M (Lehmann 2016) subtypes, and it is poorly represented in the LAR subtypes from the Burstein and Lehmann classifications. The CLDN-high subtype is highly enriched in BLIA (Burstein 2015) and BL2 (Lehmann 2016). The CLDN-low subtype is highly enriched in MES (Burstein 2015), LumA (PAM50 + CLDN-low) and BL2 (Lehmann 2016). The LAR subtype is highly enriched in LAR (Burstein 2015), LumA (PAM50 + CLDN-low) and LAR (Lehmann 2016). The LAR subtype is not present in Basal (PAM50) and BL1 (Lehmann) assignations (Fig. 5 and Table 5).

**Table 5:** Shows comparisons between Cellular classification and PAM50, Lehmann’s and Burstein’s classifications.

Basal			CLDN-High			CLDN-Low			LAR		
Burstein	N	%	Burstein	N	%	Burstein	N	%	Burstein	N	%
BLIA	104	34%	BLIA	23	58%	BLIA	11	21%	BLIA	2	2%
BLIS	149	48%	BLIS	3	1%	BLIS	3	6%	BLIS	1	1%
LAR	4	1%	LAR	4	1%	LAR	3	6%	LAR	76	84%
MES	53	17%	MES	10	25%	MES	36	68%	MES	12	13%
**PAM50 + CLDN−Low**	**N**	**%**	**PAM50 + CLDN−Low**	**N**	**%**	**PAM50 + CLDN−Low**	**N**	**%**	**PAM50 + CLDN−Low**	**N**	**%**
Basal	125	40%	Basal	13	33%	Basal	5	9%	Basal	0	0%
CLDN-Low	76	25%	CLDN-Low	9	23%	CLDN-Low	44	83%	CLDN-Low	13	14%
Her2	23	7%	Her2	6	15%	Her2	1	2%	Her2	8	9%
LumA	25	8%	LumA	7	18%	LumA	1	2%	LumA	52	57%
LumB	27	9%	LumB	4	10%	LumB	4	4%	LumB	16	18%
Normal	34	11%	Normal	1	3%	Normal	0	0%	Normal	2	2%
**TNBC4 type**	**N**	**%**	**TNBC4 type**	**N**	**%**	**TNBC4 type**	**N**	**%**	**TNBC4 type**	**N**	**%**
BL1	57	18%	BL1	8	20%	BL1	1	2%	BL1	0	0%
BL2	81	26%	BL2	29	73%	BL2	47	89%	BL2	28	31%
LAR	3	1%	LAR	0	0%	LAR	1	2%	LAR	52	57%
M	169	55%	M	3	8%	M	4	8%	M	11	12%

### Immune characteristics and previous classifications

**Figure 6:**
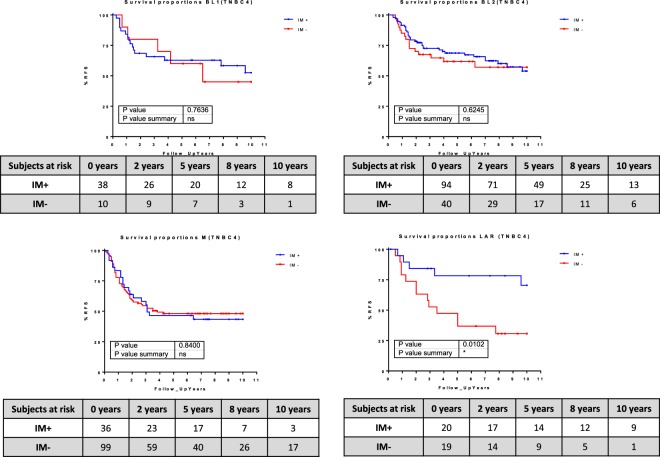
Kaplan-Meier survival curves represent the survival rate of immune-positive and immune-negative tumors in the TNBC4-type subgroups.

The Mesenchymal subtype from the TNBC4 type^7^ was highly enriched in IM- samples (148 samples of 187, 80% of all M subtype samples). Also, BL2 was enriched in IM+ samples (135 samples of 185, 72% of all BL2 subtype samples). The IM+ and IM- groups showed no prognostic value for the BL1, BL2 and M groups (Fig. 6). However, patients with IM+ tumors had better prognosis than those with IM− in the LAR group (HR, 0.2896; 95% CI 0.1125–0.7273; P < 0.05).

The IM+ and IM- subgroups were evenly distributed in the subtypes defined by PAM50 and CLDN-low, with the exception of the HER2 subtype, which was enriched in IM+ (Table 6).

**Table 6:** Shows immune characteristics in the PAM50+CLDN-low subgroups.

PAM50 + CLND-low	IM−	IM+
Basal	69 (48%)	74 (52%)
CLDN-low	62 (44%)	80 (56%)
Her2	10 (26%)	28 (74%)
LumA	43 (51%)	42 (49%)
LumB	27 (55%)	22 (57%)
Normal	24 (65%)	13 (35%)

**Figure 7:**
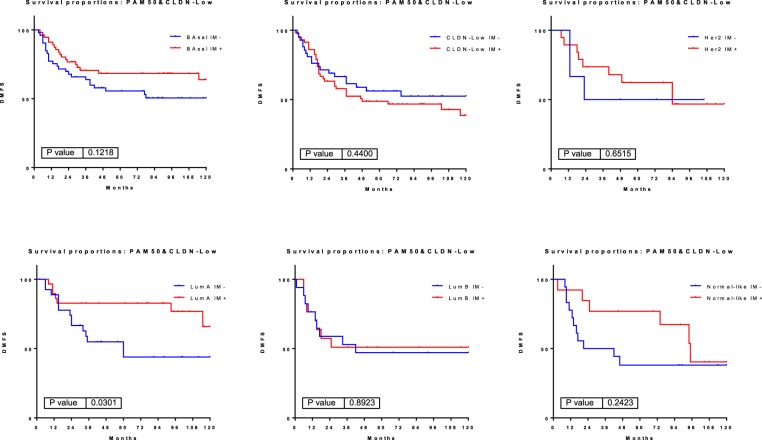
Kaplan-Meier survival curves represent the survival rate of immune-positive and immune-negative tumors in the PAM50 + CLDN-low subgroups.

LumA immune-positive tumors had a better prognosis than immune-negative tumors (HR, 2.638; 95% CI 1.098–6.341; P < 0.05). Basal Immune and normal-like immune-positive tumors also showed a trend toward a better prognosis than immunenegative, but the differences were not statistically significant. Finally CLDN-low, LumB and HER2 tumors showed no differences in prognosis related to their immune status (Fig. 7).

Finally, the Burstein subtype BLIA was highly enriched in the IM+ (106 samples of 140, 75%) and the BLIS was highly enriched in the IM- tumors (119 samples of 156, 76%).

**Figure 8:**
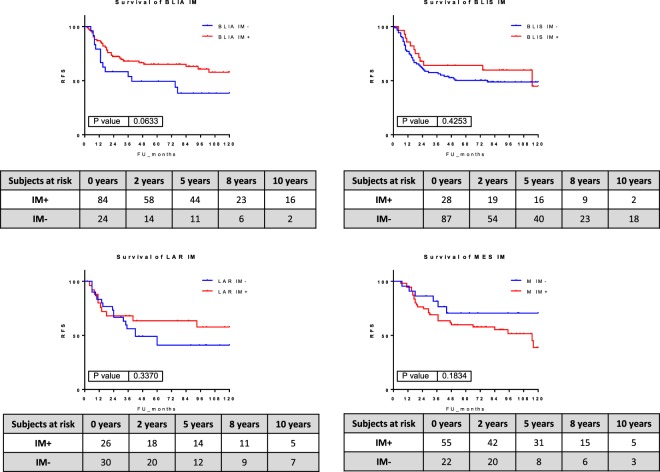
Kaplan-Meier survival curves represent the survival rate of immune-positive and immune-negative tumors in the Burstein’s subgroups.

Immune-positive and immune-negative tumors had different outcomes in each of the Burstein’s subgroups. BLIA, BLIS and LAR immune-positive tumors as well as MES immune-negative tumors had a better prognosis, although the differences were not statistically significant (Fig. 8).

### Implications of the cellular classification and the immune characteristic in response to neoadjuvant treatment

Cellular classification was transferred using genes from the basal and luminal metanodes and the CLDN-enriched functional node. Of 153 triple-negative breast cancer tumors, 79 were assigned to the basal subgroup (51%), 8 were assigned to the CLDN-high subgroup (5%), 19 were assigned to the CLDN-low subgroup (12%) and 47 were assigned to the LAR subgroup (31%). The immune characteristic was transferred using genes from the immune metanode. Some 80 samples were immune-negative (52%) and 73 samples were assigned to the immune-positive subgroup (47%) (Table 7).

**Table 7:** Shows the cellular classification and the immune characteristic in the neoadjuvant dataset.

Cellular Classification	Number	IM Characteristic	Number	%Intragroup
Basal	79 (52%)	IM−	41	52%
		IM+	38	48%
CLDN-High	8 (5%)	IM−	2	25%
		IM+	6	75%
CLDN-Low	19 (12%)	IM−	12	63%
		IM+	7	37%
LAR	47 (31%)	IM−	25	53%
		IM+	22	47%

**Figure 9:**
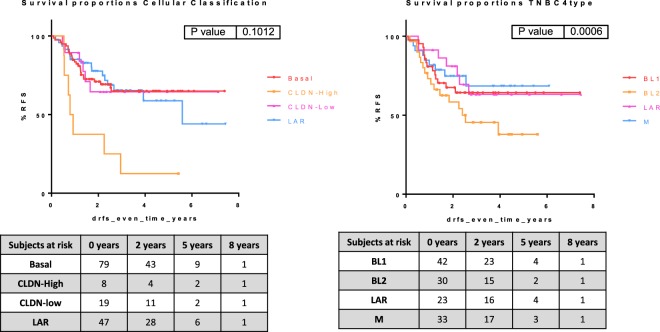
Kaplan–Meier survival curves represent the distant relapse-free survival rate of the cellular and the TNBC4-type subgroups in the GSE25066 series.

The CLDN-high subgroup presented the poorest prognosis among the cellular classification subgroups. Immune-positive tumors had a better prognosis (Fig. 9).

## Discussion

TNBC constitutes a heterogeneous disease with various molecular entities. The study of this heterogeneity has thus far not conferred significant advances in the treatment of patients. The application of probabilistic graphical models (PGMs) provides deep insight into high-throughput data^18^. In the present study, we used PGMs to unravel specific molecular information concerning various biological entities, such as the immune status or the developmental point when the breast stem cell turns carcinogenic.

Previous studies used differences in gene expression to define TNBC subtypes^3,6–8,10^. Subtypes emerged from clustering methods such as HCL or non-negative matrix factorization, which group genes around specific functions. On the contrary, we hereby applied an unsupervised analysis, without knowledge of the functions of the genes selected in each step of the process. We ultimately identified the genes involved in 26 different molecular functions, which agreed with the metagenes described by Rody *et al*.^5^. This approach provides two different classifications (immune and cellular), each related to particular genes and functions.

Once the PGM functional structure was established, we defined four subgroups: CLDN-low, CLDN-high, basal-like and LAR, agreeing with the cancer stem cell hypothesis^2,13–15^. These four groups identify the point of the differentiation process where the stem cell becomes carcinogenic: the less differentiated tumors will be CLDN-low, and the most differentiated tumors will be LAR.

Functional node activities confirm that there are differences among cellular subgroups, and some of these differences could have therapeutic utility. For example, the activity of node 19 (PPAR signaling pathway) showed meaningful differences between the CLDN-low subgroup and the other three, suggesting that PPAR-directed therapies might have a different effect on the CLDN-low subgroup. Finally, we observed that cellular subgroups had different clinical features.

On the other hand, the immune layer was described in this study as a compendium of functional nodes, each of which related to a specific immune function. However, when taking all these nodes together as a metanode we were able to establish an immune classification with prognostic value among all the series.

The immune and cellular classifications reflected unrelated biological identities. As shown in Fig. 4, the LAR and CLDN-high subgroups presented different prognoses when split by the immune layer. LAR immune-negative tumors were associated with a 30% 5-year survival rate compared with 70% in the LAR immune-positive group. The immune-based subtype might also influence the response to immunotherapy. Ongoing trials are evaluating anti-PD1 antibodies in breast cancer, particularly in triple-negative disease^24^. It would be interesting to assess the efficacy of anti-PD1 therapy in subtypes defined by immune layer.

We also compared the cellular classification with other classifications previously described^7,8,10^. LAR is overrepresented in every luminal subgroup regardless of the classification, which demonstrates that this is a homogeneous and reproducible group. Similarly, the basal cellular subgroup is overrepresented in basal subgroups across classifications. There is also a high correlation (83%) in the CLDN-low cellular groups, which confirms the existence of a CLDN-low subgroup independent of the expression of ER, PR and HER2, as previously suggested^16^.

Our results show that immune features appear across different subtypes. Interestingly, the luminal immune-positive group did much better than the luminal immune-negative group. Regardless of the classification^7,8,10^, the immune layer added prognostic information to the luminal subtypes. The immune layer had been previously defined as a separate group in these classifications, but it appears to intersect with other biological features, providing additional prognostic value.

With regard to the cellular classification, our CLDN-low cellular subgroup had an 89% concordance with the basal-like 2 Lehmann’s subgroup, which puts BL2 in the stem cell hypothesis context, suggesting that basal-like 2 tumors might be caused by early differentiated carcinogenic stem cells. The CLDN-high subgroup does not appear in other classifications, which suggests that this is an intermediate group between CLDN-low tumors (stem cell not yet expressing CLDN genes) and basal tumors. It might be difficult to draw the line between groups in this continuous, cellular differentiation-based classification, although Burnstein’s basal-like immune-active corresponded to the CLDN-high immune-negative in our classification. Regardless of the classification, there was always a luminal subgroup, one or two basal subgroups and some mesenchymal or CLDN subgroup.

Our classification could also provide some predictive information. CLDN-high tumors had a poor response to neoadjuvant chemotherapy. Much effort has been devoted to the prediction of response to chemotherapy in TNBC. Cell-free DNA^25^, tumor-infiltrating lymphocytes^26^, microRNA signatures^27^ and proteomics^28^, among others, have recently been proposed as useful methods in this regard. Further research is needed before the cellular classification described in the present paper could be considered in the selection of therapy.

This study has some limitations. The 2010 American Society of Clinical Oncology guidelines established the 1% threshold for the expression of PR and ER^29^; however, our tumor series was assessed before that date, so we cannot ensure that all the TNBC tumors fulfilled this criterion. Another limitation to our study is that the cellular classification is based on a continuum, which makes it difficult to set categories. Finally, these results should be validated in additional cohorts to evaluate the robustness of our cellular and immune classification. However, we believe that our findings serve as an important hypothesis in generating findings that can be explored in future studies.

## Conclusion

In conclusion, the use of probabilistic graphical models in TNBC suggests that there are at least two independent biological layers, cellular and immune. We propose a new way to characterize TNBC taking these two dimensions into account, and leading to the result that the luminal immune-positive subgroup had a better prognosis than the luminal immune-negative.

## Supplementary information

Sup Files 2-4

Sup. Info 1

## Data Availability

The datasets analyzed during the current study, GSE31519 [https://www.ncbi.nlm.nih.gov/geo/query/acc.cgi?acc = GSE31519], and GSE25066 [https://www.ncbi.nlm.nih.gov/geo/query/acc.cgi?acc = GSE25066], are available in the GEO Datasets repository.
