# Supplementary material for: A novel approach to triple-negative breast cancer molecular classification reveals a luminal immune-positive subgroup with good prognoses
Source: Sci Rep. 2019 Feb 7;9:1538. doi: 10.1038/s41598-018-38364-y (PMC6367406; doi:10.1038/s41598-018-38364-y)
Supplement: Supplementary file 1 — Sup Files 2-4 [file 41598_2018_38364_MOESM1_ESM.pdf]

A novel approach to triple-negative breast cancer molecular classification reveals a luminal immune-positive subgroup with good prognoses

Guillermo Prado-Vázquez[1,2], Angelo Gámez-Pozo[1,2], Lucía Trilla-Fuertes[2], Jorge M. Arevalillo[4], Andrea Zapater-Moros[1,2], María Ferrer-Gómez[1], Mariana Díaz-Almirón[3], Rocío López-Vacas[1], Hilario Navarro[4], Paloma Main[5], Jaime Feliú[6,7], Pilar Zamora[6], Enrique Espinosa[6,7], and Juan Ángel Fresno Vara[1,7,$]

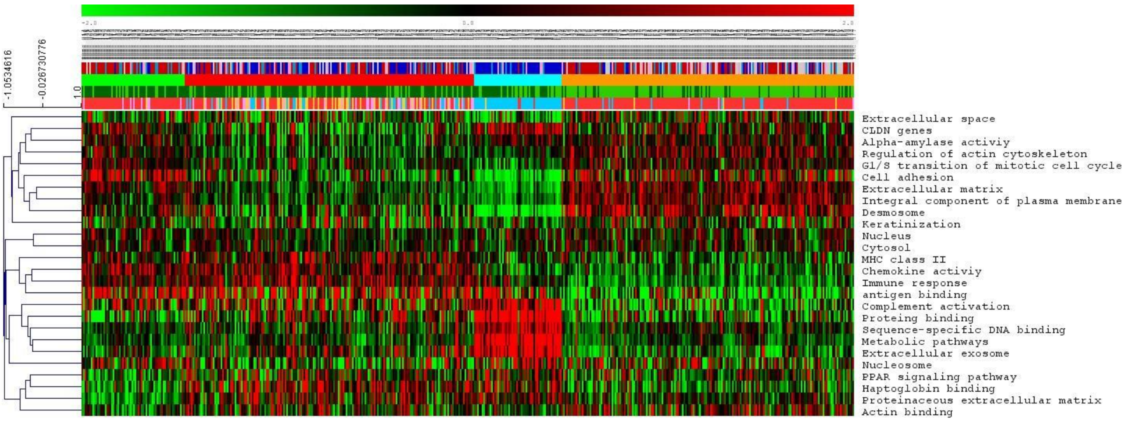

Sup. File 2 hierarchical clustering (HCL) analysis assessing functional node activity and relationships between.

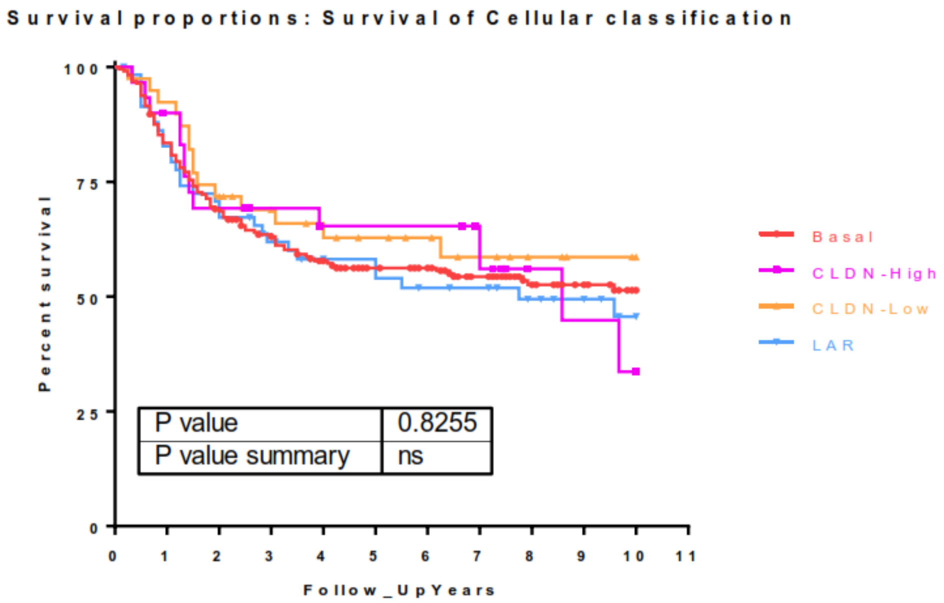

Sup. File 3 Kaplan-Meier survival curves represent the survival rate of Cellular subgroups tumors in the whole cohort.

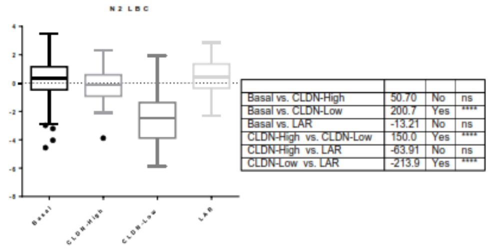

Basal vs. CLDN-High	50.70	No	ns
Basal vs. CLDN-Low	200.7	Yes	****
Basal vs. LAR	-13.21	No	ns
CLDN-High vs. CLDN-Low	150.0	Yes	****
CLDN-High vs. LAR	-63.91	No	ns
CLDN-Low vs. LAR	-213.9	Yes	****

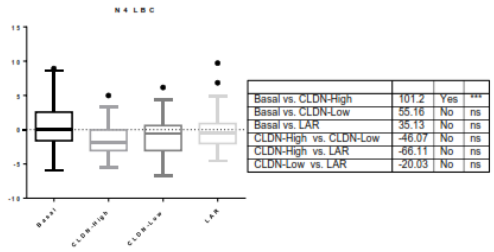

Basal vs. CLDN-High	101.2	Yes	***
Basal vs. CLDN-Low	55.16	No	ns
Basal vs. LAR	35.13	No	ns
CLDN-High vs. CLDN-Low	-46.07	No	ns
CLDN-High vs. LAR	-66.11	No	ns
CLDN-Low vs. LAR	-20.03	No	ns

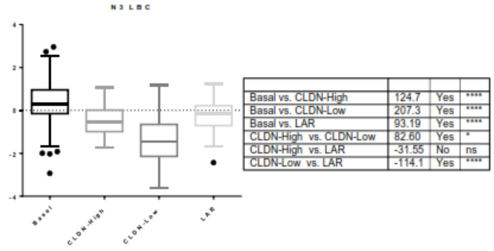

Basal vs. CLDN-High	124.7	Yes	****
Basal vs. CLDN-Low	207.3	Yes	****
Basal vs. LAR	93.19	Yes	****
CLDN-High vs. CLDN-Low	82.60	Yes	*
CLDN-High vs. LAR	-31.55	No	ns
CLDN-Low vs. LAR	-114.1	Yes	****

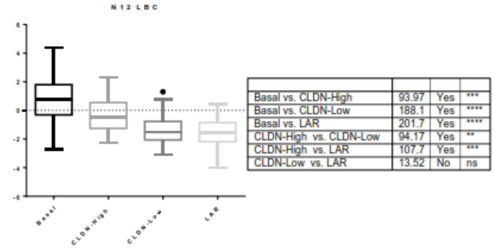

Basal vs. CLDN-High	93.97	Yes	***
Basal vs. CLDN-Low	188.1	Yes	****
Basal vs. LAR	201.7	Yes	****
CLDN-High vs. CLDN-Low	94.17	Yes	**
CLDN-High vs. LAR	107.7	Yes	***
CLDN-Low vs. LAR	13.52	No	ns

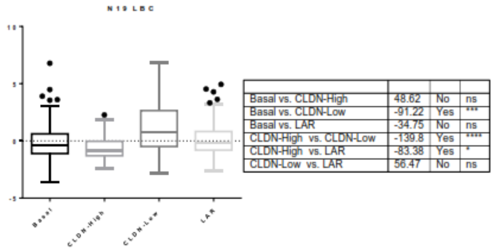

Basal vs. CLDN-High	48.62	No	ns
Basal vs. CLDN-Low	-91.22	Yes	***
Basal vs. LAR	-34.75	No	ns
CLDN-High vs. CLDN-Low	-139.8	Yes	****
CLDN-High vs. LAR	-83.38	Yes	*
CLDN-Low vs. LAR	56.47	No	ns

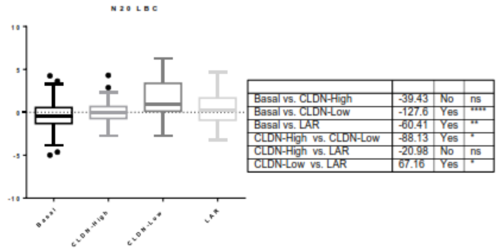

Basal vs. CLDN-High	-39.43	No	ns
Basal vs. CLDN-Low	-127.6	Yes	****
Basal vs. LAR	-60.41	Yes	**
CLDN-High vs. CLDN-Low	-88.13	Yes	*
CLDN-High vs. LAR	-20.98	No	ns
CLDN-Low vs. LAR	67.16	Yes	*

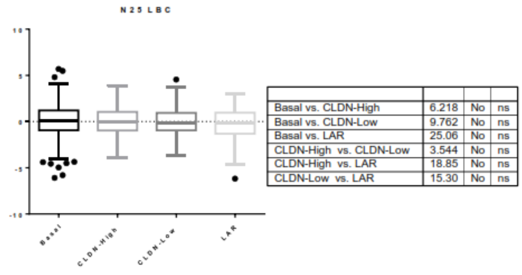

Basal vs. CLDN-High	6.218	No	ns
Basal vs. CLDN-Low	9.762	No	ns
Basal vs. LAR	25.06	No	ns
CLDN-High vs. CLDN-Low	3.544	No	ns
CLDN-High vs. LAR	18.85	No	ns
CLDN-Low vs. LAR	15.30	No	ns

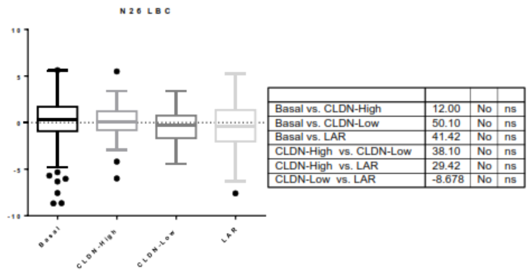

Basal vs. CLDN-High	12.00	No	ns
Basal vs. CLDN-Low	50.10	No	ns
Basal vs. LAR	41.42	No	ns
CLDN-High vs. CLDN-Low	38.10	No	ns
CLDN-High vs. LAR	29.42	No	ns
CLDN-Low vs. LAR	-8.678	No	ns

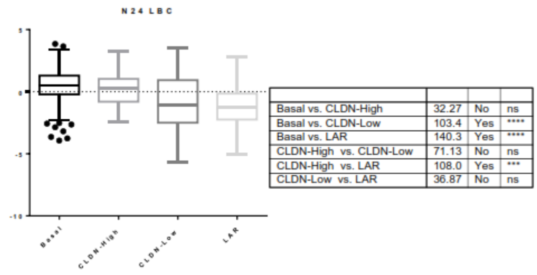

Basal vs. CLDN-High	32.27	No	ns
Basal vs. CLDN-Low	103.4	Yes	****
Basal vs. LAR	140.3	Yes	****
CLDN-High vs. CLDN-Low	71.13	No	ns
CLDN-High vs. LAR	108.0	Yes	***
CLDN-Low vs. LAR	36.87	No	ns

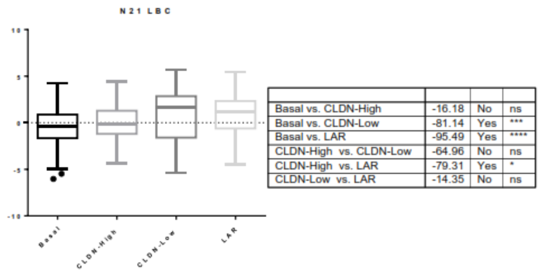

Basal vs. CLDN-High	-16.18	No	ns
Basal vs. CLDN-Low	-81.14	Yes	***
Basal vs. LAR	-95.49	Yes	****
CLDN-High vs. CLDN-Low	-64.96	No	ns
CLDN-High vs. LAR	-79.31	Yes	*
CLDN-Low vs. LAR	-14.35	No	ns

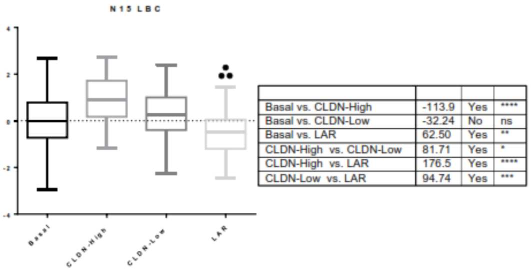

Basal vs. CLDN-High	-113.9	Yes	****
Basal vs. CLDN-Low	-32.24	No	ns
Basal vs. LAR	62.50	Yes	**
CLDN-High vs. CLDN-Low	81.71	Yes	*
CLDN-High vs. LAR	176.5	Yes	****
CLDN-Low vs. LAR	94.74	Yes	***

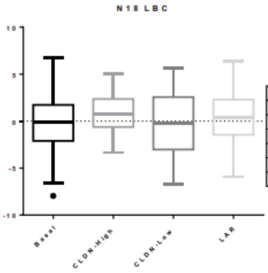

Basal vs. CLDN-High	-58.85	No	ns
Basal vs. CLDN-Low	2.991	No	ns
Basal vs. LAR	-28.00	No	ns
CLDN-High vs. CLDN-Low	61.84	No	ns
CLDN-High vs. LAR	30.85	No	ns
CLDN-Low vs. LAR	-31.00	No	ns

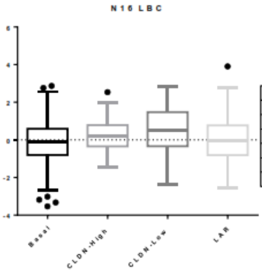

Basal vs. CLDN-High	-45.79	No	ns
Basal vs. CLDN-Low	-68.23	Yes	**
Basal vs. LAR	-6.574	No	ns
CLDN-High vs. CLDN-Low	-22.44	No	ns
CLDN-High vs. LAR	39.21	No	ns
CLDN-Low vs. LAR	61.66	No	ns

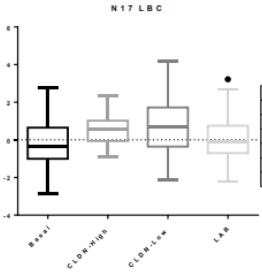

Basal vs. CLDN-High	-96.54	Yes	***
Basal vs. CLDN-Low	-99.15	Yes	****
Basal vs. LAR	-19.21	No	ns
CLDN-High vs. CLDN-Low	-2.610	No	ns
CLDN-High vs. LAR	77.34	Yes	*
CLDN-Low vs. LAR	79.95	Yes	**

Sup file 4. The activity of the main functional nodes was assessed in each cellular group by boxplot analysis.
